# Mulberry (*Morus alba* L.) Leaf Extract and 1-Deoxynojirimycin Improve Skeletal Muscle Insulin Resistance via the Activation of IRS-1/PI3K/Akt Pathway in *db*/*db* Mice

**DOI:** 10.3390/life12101630

**Published:** 2022-10-18

**Authors:** Chae-Won Kang, Miey Park, Hae-Jeung Lee

**Affiliations:** 1 Institute for Aging and Clinical Nutrition Research, Gachon University, Seongnam 13120, Gyeonggi-do, Korea; 2 Department of Food and Nutrition, College of BioNano Technology, Gachon University, Seongnam 13120, Gyeonggi-do, Korea

**Keywords:** *Morus alba* L., 1-deoxynojirimycin, *db*/*db* mice, type 2 diabetes mellitus, skeletal muscle, IRS-1/PI3K/Akt pathway

## Abstract

Mulberry (*Morus alba* L.) leaves have been used to lower blood glucose in patients with diabetes. We evaluated the effects of mulberry leaves extract (MLE) and 1-deoxynojirimycin (1-DNJ) in improving insulin resistance through the activation of the IRS-1/PI3K/Akt pathway in the skeletal muscle of *db*/*db* mice. Histological analysis revealed an amelioration of muscle deformation and increased muscle fiber size. MLE and 1-DNJ positively raised the protein expression of related glucose uptake and increased the translocation of glucose transporter type 4 (GLUT4) to the membrane. Furthermore, MLE and 1-DNJ activated the IRS-1/PI3K/Akt pathway in the skeletal muscle and, subsequently, modulated the protein levels of glycogen synthase kinase-3beta (GSK-3β) and glycogen synthase (GS), leading to elevated muscle glycogen content. These findings suggest that MLE and 1-DNJ supplementation improves insulin resistance by modulating the insulin signaling pathway in the skeletal muscle of *db*/*db* mice.

## 1. Introduction

Type 2 diabetes mellitus (T2DM) is a metabolic disorder, in which insulin-mediated glucose uptake in peripheral tissues, primarily the liver, muscle, and adipose tissues, decreases due to insulin resistance (IR) [1]. The development of IR is caused by the impaired biologic response to insulin stimulation in the target tissues, resulting in hyperglycemia, hyperinsulinemia, dyslipidemia, obesity, and hypertension [2].

The skeletal muscle plays an important role in the maintenance of postprandial glucose homeostasis, and is the leading contributor to whole-body energy expenditure [3]. In the skeletal muscle, glucose metabolism is mainly regulated by insulin, which exerts its metabolic actions by activating the signaling cascade. However, IR reduces the ability of the skeletal muscle to take up and utilize glucose [4,5]. Moreover, impaired-glucose utilization is associated with metabolic dysregulation of the skeletal muscle, leading to muscle damage and a reduction in muscle mass and strength, thereby increasing the risk of sarcopenia [6,7].

The insulin receptor substrate 1 (IRS-1) is the most critical signaling protein in the muscle, which activates intracellular signaling cascades, thereby facilitating the cellular response to insulin [8]. The activation of IRS-1 stimulates the p85 regulatory subunit, phosphatidylinositol 3-kinase (PI3K), which is associated with an extraordinarily diverse group of cellular functions [9]. PI3K phosphorylation activates downstream protein kinase B (Akt), the key signaling molecule in regulating multiple cellular processes such as glucose metabolism, apoptosis, and cell proliferation [10]. The IRS-1/PI3K/Akt pathway is one of the most important signaling pathways that regulate insulin resistance and glucose homeostasis, and its activation is essential for normal insulin-mediated glucose metabolism in the skeletal muscle [11,12]. The translocation of the glucose transporter type 4 (GLUT4) from the intracellular vesicles to the plasma membrane is an essential mechanism in the regulation of glucose uptake, and is mediated via the insulin signaling pathway [13,14]. Peroxisome proliferator-activated receptor gamma (PPARγ) plays an essential role in the regulation of glucose metabolism in insulin-resistant conditions [15]. The peroxisome proliferator-activated receptor-gamma coactivator 1 alpha (PGC-1α) is a significant regulator of skeletal muscle mitochondrial function, and can enhance GLUT4 translocation [16]. The glycogen synthase kinase-3beta (GSK-3β) in insulin resistance inactivates glycogen synthase (GS) phosphorylation, resulting in a decrease in glycogen synthesis [17].

Mulberry leaf (*Morus alba* L.) contains large amounts of bioactive compounds, including polyphenols and flavonoids, with various biological activities, including antioxidant and anti-inflammatory effects [18]. Furthermore, numerous studies have shown that mulberry leaf extract (MLE) displays an ability to lower blood glucose levels in patients with diabetes [19]. 1-Deoxynojirimycin (1-DNJ) is abundant in mulberry leaves and is a well-known alpha-glucosidase inhibitor [20]. Huang et al. have previously reported that the purified 1-DNJ treatment appeared to improve insulin sensitivity in streptozotocin-induced diabetic rats [21]. Several studies have been performed on the various biological activities of another mulberry, but few studies are available concerning the molecular mechanisms of MLE or 1-DNJ in ameliorating insulin resistance in the skeletal muscles of *db*/*db* mice. 

Therefore, our study aimed to investigate whether MLE or 1-DNJ supplementation could improve muscle insulin resistance by activating the IRS-1/PI3K/Akt pathway in the skeletal muscles of diabetic *db*/*db* mice. 

## 2. Materials and Methods

### 2.1. Reagents and Chemicals

Analytical-grade acetonitrile, methanol, and water were obtained from Thermo Fisher Scientific (Pittsburgh, PA, USA). Pyridine, methoxyamine hydrochloride, *N*-methyl-*N*-(trimethylsilyl) trifluoroacetamide (MSTFA), and standard compounds were purchased from Sigma Chemical Co., (St. Louis, MO, USA). The 1-Deoxynojirimycin (1-DNJ) from root barks of *Morus alba* L. was obtained from ChemFaces (ChemFaces Biochemical Co., Ltd., Wuhan, China). In this study, 1-DNJ powder was dissolved in phosphate-buffered saline (PBS), and the stock was stored at −20 °C.

### 2.2. Mulberry Leaf Extract (MLE) Powder Preparation for Metabolite Analysis

Mulberry leaf was purchased from the Shin Young-deok Sericulture Association (Yeongdeok-gun, Gyeongbuk, Korea). Fresh samples of mulberry leaf were washed in tap water and then rewashed with distilled water. The leaf was dried in an oven at 50 °C for 9 h, and crushed into crumbs. The crushed mulberry leaves (40 g) were then suspended in 800 mL of distilled water, equivalent to 20 times the mulberry leaf’s weight, at 90 °C for 4 h. The mulberry leaf extract (MLE) was filtered through qualitative 5 μm filter paper and evaporated using a rotary evaporator at 60 °C. The concentrated samples were pre-frozen and then lyophilized at −80 °C for 72 h. For metabolite analysis, lyophilized MLE powder (100 mg) was extracted in 80% methanol (1 mL) containing an internal standard solution (10 μL; 2-chloro-phenylalanine, 1 mg/mL in water) using an MM400 mixer mill (Retsch^®^, Haan, Germany) at a frequency of 30 s^−1^ for 10 min, followed by 10 min of sonification. Subsequently, the extracted samples were centrifuged at 15,000× *g* rpm for 10 min at 4 °C. After centrifugation, the supernatants were filtered through 0.2-μm polytetrafluoroethylene (PTFE) filters (Chromdisc, Daegu, Korea). The filtered supernatants were completely evaporated using a speed vacuum concentrator (Biotron, Seoul, Korea) to obtain a final concentration of 10 mg/mL for mass spectrometry (MS) analysis.

### 2.3. Gas Chromatography-Time of Flight-Mass Spectrometry Analysis (GC-TOF-MS)

GC-TOF-MS analysis was performed as described by our previous study [22]. Oximation was achieved by adding 50 μL of methoxyamine hydrochloride (20 mg/mL in pyridine) to each lyophilized sample for GC-TOF-MS analysis, and the mixture was incubated for 90 min at 30 °C. After incubation, the silylation was performed by adding 50 μL of MSTFA for 30 min at 37 °C. Then, the derivatized sample (1 μL) was injected into the GC-TOF-MS instrument at a split ratio of 10:1.

### 2.4. Ultrahigh Performance Liquid Chromatography-Linear Trap Quadrupole-Orbitrap-Mass Spectrometry (UHPLC-LTQ-Orbitrap-MS/MS) Analysis

UHPLC-LTQ-Orbitrap-MS analysis was processed as described by a previous study [22]. For UHPLC-LTQ-Orbitrap-MS analysis, the lyophilized sample was re-dissolved in 80% methanol and then conducted. The injection volume for all samples was 5 μL into a UHPLC system equipped with a Vanquish binary pump H system (Thermo Fisher Scientific, Waltham, MA, USA). The separation of metabolites was carried out on a reversed-phase Syncronis C18 UHPLC column (1.7 μm size; Thermo Fisher Scientific, Waltham, MA, USA). The mobile phase consisted of (A) water with 0.1% formic acid, and (B) acetonitrile with 0.1% formic acid. The spectra were acquired in the mass range of 100–1000 *m*/*z*.

### 2.5. Experimental Animals and Diets

Eight-week-old male *db*/*db* mice (BKS.Cg-*Dock*7*m* +/+ *Leprdb*/*J* mice, homozygote) and *db*/*m* mice (C57BL/6J mice, heterozygotes) were purchased from Jackson Laboratories (Sacramento, CA, USA). All mice were housed in a temperature-controlled room at 20–25 °C and 50–60% humidity, and were maintained under a 12-h light/dark cycle. The mice were acclimatized to the experimental facility for 2 weeks. All the mice were randomly divided into 7 groups with six mice in each group and treated for 35 days as follows: (1) *db*/*m* + saline (N+); (2) *db*/*db* + saline (NC); (3) *db*/*db* + metformin (200 mg/kg/day) (PC); (4) *db*/*db* + low-dose MLE (200 mg/kg/day) (M200); (5) *db*/*db* + high-dose MLE (500 mg/kg/day) (M500); (6) *db*/*db* + 1-DNJ (40 mM/kg/day) (1-DNJ); (7) *db*/*db* + high-dose MLE (500 mg/kg/day) + Akt inhibitor (30 mM/kg/day). All the groups received an equal volume by oral gavage. Body weight was monitored twice a week. All animal experiments were approved by the Gachon University for the care and use of laboratory animals (GIACUC-R2020012). 

### 2.6. Fasting Blood Glucose Levels (FBGLs), Oral Glucose Tolerance Test (OGTT), and Insulin Tolerance Test (ITT)

The fasting blood glucose levels were assessed every 2 days using a blood glucose test meter (Roche Accu-chek, Mannheim, Germany) with blood drawn from the tail vein. Mice were subjected to glucose tolerance and insulin tolerance tests on days 21 and 35. For the oral glucose tolerance test (OGTT), mice were fasted for 12 h and administered glucose orally at a dose of 2 g/kg body weight. After glucose loading, blood glucose was measured at 15, 30, 60, and 120 min. For the insulin tolerance test (ITT), mice were fasted for 3 h and intraperitoneally injected with human biosynthetic insulin at a dose of 1 U/kg body weight. After insulin loading, blood glucose was measured at 15, 30, and 60 min. The area under the curve (AUC) was statistically analyzed. 

### 2.7. Serum Biochemical Analysis

At the end of the experiment, the mice were fasted overnight and sacrificed by CO_2_ asphyxiation. Blood was collected from the mice, stored at room temperature for 30 min, and centrifuged at 3000 rpm at 4 °C for 10 min to obtain serum. The concentrations of serum triglyceride (TG) and total cholesterol (TC) were assayed enzymatically using commercial kits (Asan pharms, Co., Seoul, Korea). Serum insulin content was detected using the mouse insulin ELISA kit (FUJIFILM, Co., Santa Clara, CA, USA). The insulin concentrations were expressed as mU/mL. Insulin resistance was measured with the homeostasis model assessment of insulin resistance (HOMA-IR), and was calculated using the formula: HOMA-IR = {Fasting glucose (mg/dl) × Fasting insulin (μU/L)}/405 [21]. 

### 2.8. Measurement of Glycogen Content in the Skeletal Muscle

The muscle glycogen content was measured using the procedure described in the colorimetric glycogen assay kit (Abcam, Cambridge, UK). The frozen skeletal muscle was resuspended in 200 μL double distilled water (ddH_2_O) on ice and homogenized. The homogenates were boiled (10 min) to inactivate enzymes, and centrifuged (18,000× *g*) to remove insoluble material. The glycogen content was measured with the supernatant. The optical density (OD) value of each group was determined at 570 nm, and the glycogen content was calculated using a standard glycogen curve. 

### 2.9. Extraction of Membrane Protein from Skeletal Muscle

The extraction of the membrane protein from the skeletal muscle tissue was performed using a membrane protein extraction kit (Thermo Fisher Scientific, Waltham, MA, USA) according to the manufacturer’s instructions. The skeletal muscle samples were washed with phosphate-buffered saline (PBS). After homogenizing the samples with a permeabilization buffer, they were incubated at 4 °C for 10 min. The homogenates were centrifuged for 15 min at 16,000× *g*, 4 °C to remove the nucleus and unfragmented cells. The pellet was resuspended in the solubilization buffer and constantly mixed for 30 min. The lysate was centrifuged (4 °C, 16,000× *g*, 15 min) to obtain a supernatant containing the solubilized membrane, which was stored at −80 °C.

### 2.10. Western Blot Analysis

The skeletal muscle tissues were homogenized in ice-cold lysis buffer (PRO-PREP; iNtRON Biotechnology, Seoul, Korea) with a 1% phosphatase inhibitor (Thermo Scientific, Waltham, MA, USA) to extract the proteins. Then, the lysed homogenates were centrifuged at 13,000 rpm for 10 min at 4 °C, and the supernatant was collected as the lysate. The proteins (40 μg) were separated by 10% sodium dodecyl sulfate-polyacrylamide gel electrophoresis (SDS-PAGE) and transferred onto polyvinylidene fluoride (PVDF) membranes. The membranes were blocked in 5% skim milk at room temperature for 1 h and probed with primary antibodies against GLUT4, PPARγ, PGC-1α, IRS-1, p-PI3K, PI3K, p-Akt, Akt, p-GSK-3β, GSK-3β, p-GS, GS, α-tubulin, and Na+-K+-ATPaseα1. The level of α-tubulin was estimated for equal loading of the sample. Normalization of the membrane GLUT4 (mGLUT4) expression was carried out using Na+-K+-ATPaseα1 as control. Membranes were washed three times with Tris-buffered saline Tween 20 (TBST) and incubated for 1 h at room temperature with the appropriate horseradish peroxidase-conjugated secondary antibodies. After three additional TBST washes, band detection was visualized by enhanced chemiluminescence (ECL) detection reagents and the LAS 500 imager (GE Healthcare Bio-Sciences AB, Uppsala, Sweden). 

### 2.11. Hematoxylin and Eosin (H&E) Staining

The skeletal muscle tissues were fixed in 10% formalin, embedded in paraffin, and stained with hematoxylin and eosin (H&E). The histological changes were observed and photographed with an Olympus Provis AX 70 microscope (Olympus, Tokyo, Japan). Images were captured using a Nikon DS-Ri2 camera (Nikon, Tokyo, Japan) with the NIS Elements BR 4.50.00 software (Tokyo, Japan), and muscle fiber size was measured using Image J software (n = 3 animals per group).

### 2.12. Data Analysis

All data are presented as mean ± standard deviation (SD). Statistical analysis was performed using a one-way analysis of variance (ANOVA) followed by Tukey’s post hoc test using the GraphPad Prism 5.03 for Windows software (GraphPad Software, San Diego, CA, USA). Values of *p* < 0.05 were considered to indicate a statistically significant difference for all data analyses. MS-based multivariate statistical analyses were conducted as previously described in our study [22]. Based on various databases, the identification of selected metabolites was confirmed by comparison of the standard compounds retention time, MS^n^ fragments pattern, and mass spectrum (*m*/*z*). Furthermore, we verified the MS spectrum data for selected metabolites with an available database, including the National Institute of Standards and Technology (NIST) database (Version 2.0, 2011, FairCom, Gaithersburg, MD, USA), Wiley 9, and the Human Metabolome Database (HMDB; http://www.hmdb.ca/ accessed on 1 February 2022). Statistical analysis was performed using PASW statistics (IBM SPSS Inc., Chicago, IL, USA). 

## 3. Results

### 3.1. Metabolites Identified through GC-TOF-MS and UHPLC-LTQ-Orbitrap-MS Analyses 

GC-TOF-MS and UHPLC-LTQ-Orbitrap-MS analyses were performed to explore the active compounds in lyophilized MLE powder. The representative chromatograms of lyophilized MLE powder in the ion modes are presented in Figure S1. In GC-TOF-MS analysis a VIP value above 0.7 and *p*-value below 0.05 was selected, a total of 28 metabolites, including 8 amino acids, 10 of which are sugar and their derivatives, 2 fatty acids, 8 various metabolites, and 16 non-identified compounds were detected (Table S1). In UHPLC-LTQ-Orbitrap-MS analyses, a total of 28 metabolites, including 2 carboxylic acids, 2 hydroxybenzoic acids, 5 phenolic acids, 11 flavonols, 6 lysophospholipids, and 2 various metabolites, were identified (Table S2). 

### 3.2. Effects of MLE or 1-DNJ on Body Weight and FBGLs in Diabetic db/db Mice

**Figure 1:**
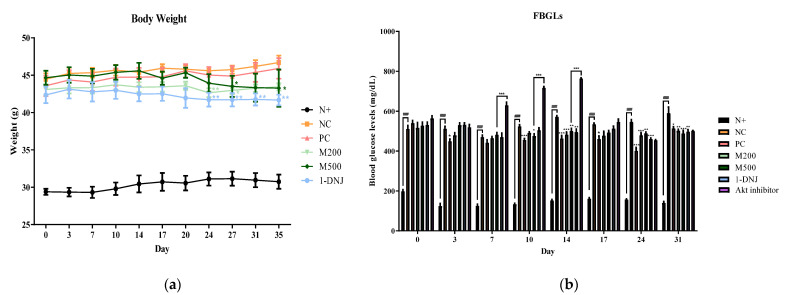
Effects of MLE or 1-DNJ on weight gain and blood glucose levels in diabetic *db*/*db* mice. (**a**) Body weights during experimental stage. (**b**) Fasting blood glucose levels. N+, *db*/*m* + saline; NC, *db*/*db* + saline; PC, *db*/*db* + metformin 200 mg/kg/day; M200, *db*/*db* + MLE 200 mg/kg/day; M500, *db*/ *db* + MLE 500 mg/kg/day; 1-DNJ, *db*/*db* + 1-DNJ 40 mM/kg/day; Akt inhibitor, *db*/*db* + MLE 500 mg/kg/day + Akt inhibitor 30 mM/kg/day. Values are expressed as mean ± SE. ### *p* < 0.001 vs. N+; * *p* < 0.05, ** *p* < 0.01, *** *p* < 0.001 vs. NC.

Throughout the experiment, the body weights of all the experimental mice were monitored twice a week (Figure 1a). Although there was no significant difference in the first week of treatment, the weights of the *db*/*db* mice treated with MLE or 1-DNJ started to reduce significantly compared to the NC group from the 24th day of treatment. At the end of the administration, the body weights of the MLE- or 1-DNJ-treated groups had significantly decreased compared to the NC group. Furthermore, the initial fasting blood glucose levels in all the *db*/*db* mice were higher than those of the *db*/*m* mice, and the N+ group was stable and unchanged (Figure 1b). The fasting blood-glucose-lowering effects of MLE or 1-DNJ were comparable to that of metformin. The results indicate that dietary MLE or 1-DNJ can reduce the body weight and FBGLs of diabetic mice. 

### 3.3. Effects of MLE or 1-DNJ on Oral Glucose Tolerance and Insulin Tolerance in Diabetic db/db Mice

**Figure 2:**
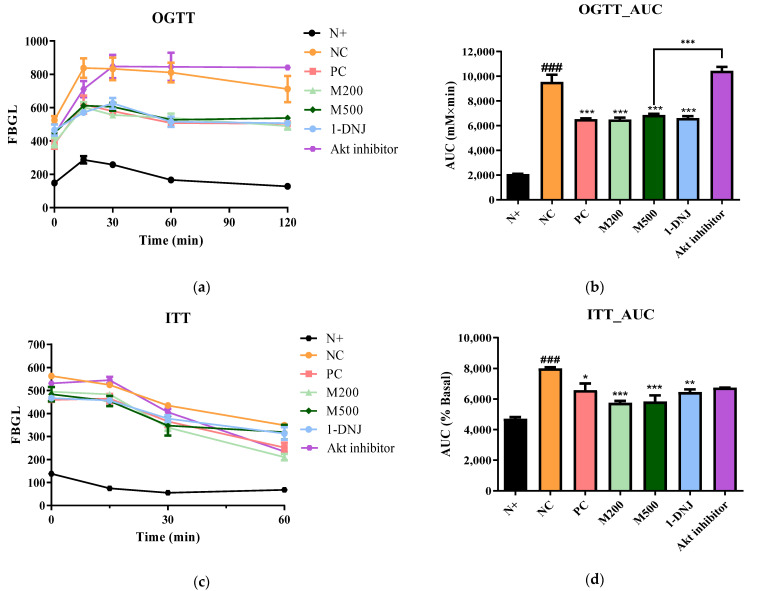
Effects of MLE or 1-DNJ on the parameters of glucose tolerance and insulin tolerance in diabetic *db*/*db* mice. (**a**) OGTT. (**b**) AUC of OGTT. (**c**) ITT. (**d**) AUC of ITT. N+, *db*/*m* + saline; NC, *db*/*db* + saline; PC, *db*/*db* + metformin 200 mg/kg/day; M200, *db*/*db* + MLE 200 mg/kg/day; M500, *db*/*db* + MLE 500 mg/kg/day; 1-DNJ, *db*/*db* + 1-DNJ 40 mM/kg/day; Akt inhibitor, *db*/*db* + MLE 500 mg/kg/day + Akt inhibitor 30 mM/kg/day. Values are expressed as mean ± SE. ### *p* < 0.001 vs. N+; * *p* < 0.05, ** *p* < 0.01, *** *p* < 0.001 vs. NC.

After the administration of MLE or 1-DNJ for 21 days, we performed OGTT to evaluate blood glucose homeostasis (Figure 2a). The blood glucose levels in all the groups peaked at 15 min after glucose administration, and the levels gradually declined as time passed. Compared with the *db*/*m* mice, all *db*/*db* mice showed a significantly stronger hyperglycemic response; however, the supplementation of MLE or 1-DNJ substantially lowered blood glucose levels compared to the NC group. Similarly, the AUCs of the OGTT performed on the MLE or 1-DNJ groups decreased compared to those of the NC group (Figure 2b). At 35 days of MLE or 1-DNJ treatment, insulin resistance was examined using the ITT method (Figure 2c). The blood glucose levels in all groups exhibited a gradual reduction, and then significantly decreased at 60 min after an intraperitoneal insulin injection. As shown in Figure 2d, the AUCs of the MLE or 1-DNJ groups were significantly reduced compared to the NC group. These data indicate that MLE or 1-DNJ may ameliorate glucose and insulin tolerance in diabetic *db*/*db* mice.

### 3.4. Effects of MLE or 1-DNJ on Serum Biochemical Parameters in Diabetic db/db Mice

**Figure 3:**
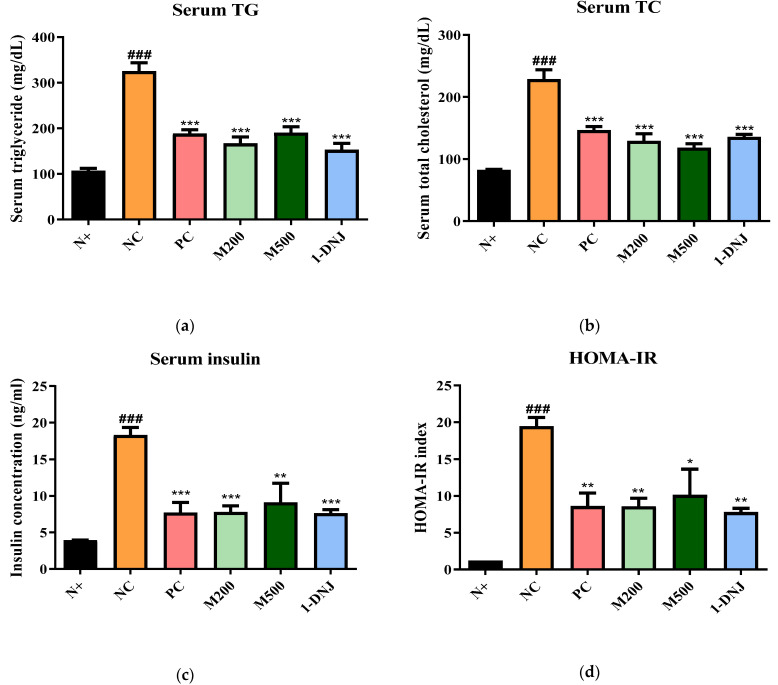
Effects of MLE or 1-DNJ on the serum lipid profiles, insulin concentration, and homeostasis model assessment of insulin resistance (HOMA-IR) in diabetic *db*/*db* mice. (**a**) Serum triglyceride (TG) levels. (**b**) Serum total cholesterol (TC) levels. (**c**) Serum insulin concentration. (**d**) HOMA-IR was calculated as (fasting serum glucose * fasting serum insulin)/22.5. N+, *db*/*m* + saline; NC, *db*/*db* + saline; PC, *db*/*db* + metformin 200 mg/kg/day; M200, *db*/*db* + MLE 200 mg/kg/day; M500, *db*/*db* + MLE 500 mg/kg/day; 1-DNJ, *db*/*db* + 1-DNJ 40mM/kg/day. Values are expressed as mean ± SE. ### *p* < 0.001 vs. N+; * *p* < 0.05, ** *p* < 0.01, *** *p* < 0.001 vs. NC.

Compared to the N+ group, the serum TG and TC levels in the NC group markedly increased. After 35 days of treatment, the serum TG and TC levels in the MLE or 1-DNJ groups decreased compared with the NC group (Figure 3a,b). Hyperinsulinemia is a well-known feature of T2DM [2]. A high serum insulin concentration was observed in the NC group compared with the N+ group, which was reversed after the administration of MLE or 1-DNJ (Figure 3c). Consistent with the changes in the serum insulin concentration, MLE or 1-DNJ supplementation significantly decreased the HOMA-IR index (Figure 3d).

### 3.5. Effects of MLE or 1-DNJ on the Pathology of Diabetic Skeletal Muscles

**Figure 4:**
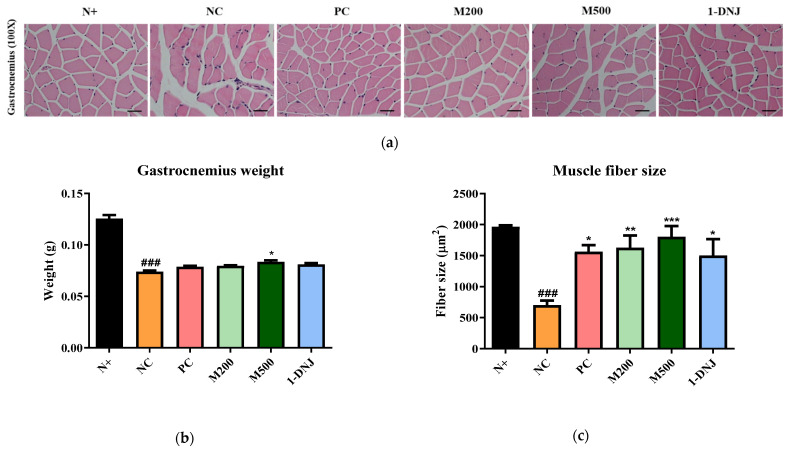
Effects of MLE or 1-DNJ on the pathology of diabetic skeletal muscle. (**a**) H&E staining of skeletal muscle in *db*/*db* mice. The bar indicates 100 μm. Magnification: 100×. (**b**) Gastrocnemius weight. (**c**) Muscle fiber size. N+, *db*/*m* + saline; NC, *db*/*db* + saline; PC, *db*/*db* + metformin 200 mg/kg/day; M200, *db*/*db* + MLE 200 mg/kg/day; M500, *db*/*db* + MLE 500 mg/kg/day; 1-DNJ, *db*/*db* + 1-DNJ 40mM/kg/day. Values are expressed as mean ± SE. ### *p* < 0.001 vs. N+; * *p* < 0.05, ** *p* < 0.01, *** *p* < 0.001 vs. NC.

H&E staining showed skeletal muscle fiber deformation and cell disorders in the NC group compared with the N+ group, whereas the supplementation with MLE or 1-DNJ partially improved the pathological changes of IR (Figure 4a). As shown in Figure 4b, the weight of the gastrocnemius muscle in all the *db*/*db* mice remarkably decreased compared with the N+ group, and there was no significant difference between the treatment groups, but the M500 group showed an increase compared to the NC group. Furthermore, the muscle fiber size in the NC group was significantly reduced compared with the N+ group, whereas the supplementation with MLE or 1-DNJ significantly increased the muscle fiber diameter (Figure 4c).

### 3.6. Effects of MLE or 1-DNJ on Glucose Metabolism in Diabetic Skeletal Muscle

**Figure 5:**
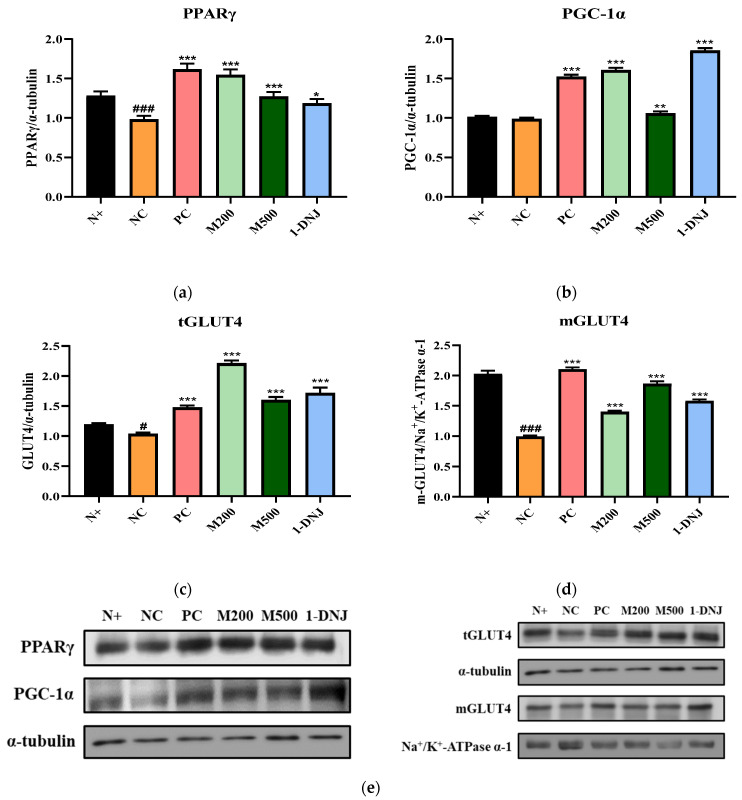
Effects of MLE or 1-DNJ on glucose metabolism of diabetic skeletal muscle. (**a**) PPARγ. (**b**) PGC-1α. (**c**) tGLUT4. (**d**) mGLUT4. (**e**) Western blot analysis for PPARγ, PGC-1α, tGLUT4, and mGLUT4 protein expression in diabetic skeletal muscle. N+, *db*/*m* + saline; NC, *db*/*db* + saline; PC, *db*/*db* + metformin 200 mg/kg/day; M200, *db*/*db* + MLE 200 mg/kg/day; M500, *db*/*db* + MLE 500 mg/kg/day; 1-DNJ, *db*/*db* + 1-DNJ 40 mM/kg/day. Values are expressed as mean ± SE. # *p* < 0.05, ### *p* < 0.001 vs. N+; * *p* < 0.05, ** *p* < 0.01, *** *p* < 0.001 vs. NC.

To investigate the effects of MLE or 1-DNJ on glucose metabolism, we evaluated the PPARγ, PGC-1α, and GLUT4 protein expression levels by western blot analysis. As shown in Figure 5a,b, the protein expression of PPARγ and PGC-1α involved in glucose metabolism in the skeletal muscle of the *db*/*db* mice was increased by the MLE or 1-DNJ treatment compared with those of the NC group. The total GLUT4 (tGLUT4) protein and translocation of membrane GLUT4 (mGLUT4) protein in the skeletal muscle of the *db*/*db* mice were down-regulated in the NC group compared to the N+ group (Figure 5c,d). However, MLE or 1-DNJ treatment promoted tGLUT4 protein expression and the translocation of the GLUT4 from the cytoplasm to the plasma membrane in *db*/*db* mice. These results suggest that the MLE or 1-DNJ treatment improved hyperglycemia through the up-regulation of glucose metabolism.

### 3.7. Effects of MLE or 1-DNJ on the IRS-1/PI3K/Akt Signaling Pathway in Diabetic Skeletal Muscle

**Figure 6:**
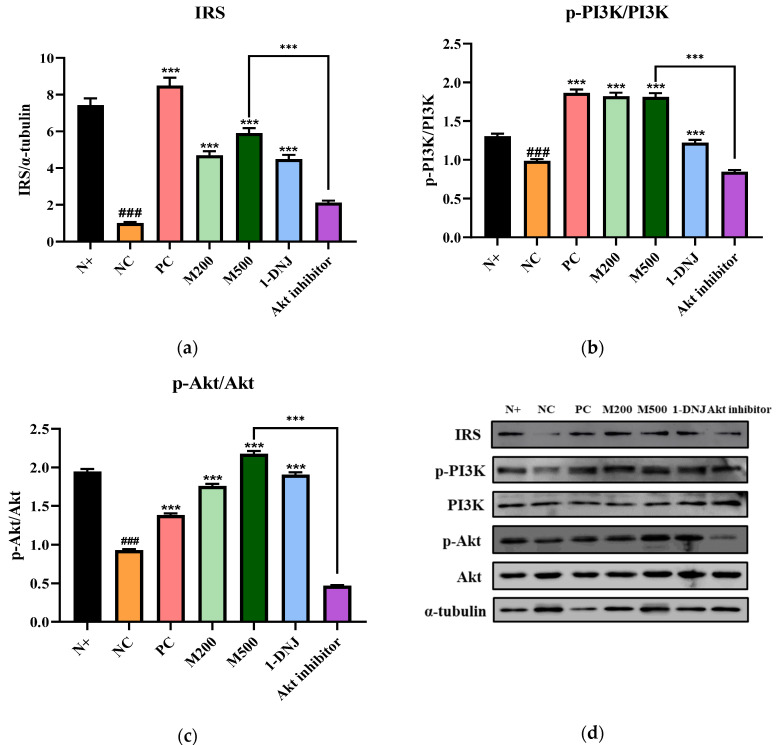
Effects of MLE or 1-DNJ on IRS-1/PI3K/Akt insulin signaling pathway of diabetic skeletal muscle. (**a**) IRS-1. (**b**) p-PI3K/PI3K. (**c**) p-Akt/Akt. (**d**) Western blot analysis for IRS-1, p-PI3K, PI3K, p-Akt, and Akt protein expression in diabetic skeletal muscle. N+, *db*/*m* + saline; NC, *db*/*db* + saline; PC, *db*/*db* + metformin 200 mg/kg/day; M200, *db*/*db* + MLE 200 mg/kg/day; M500, *db*/*db* + MLE 500 mg/kg/day; 1-DNJ, *db*/*db* + 1-DNJ 40 mM/kg/day; Akt inhibitor, *db*/*db* + MLE 500 mg/kg/day + Akt inhibitor 30 mM/kg/day. Values are expressed as mean ± SE. ### *p* < 0.001 vs. N+; *** *p* < 0.001 vs. NC.

We assessed the effects of MLE or 1-DNJ on insulin resistance in mice. The expression of proteins involved in the insulin signaling pathway in the diabetic skeletal muscle was analyzed (Figure 6a–d). IRS-1, p-PI3K, and p-Akt protein expression levels significantly decreased in the NC and Akt inhibitor group compared to the N+ group, suggesting impaired insulin signaling cascades. However, the MLE or 1-DNJ treatment markedly mitigated the expression levels of these proteins without affecting the expression levels of total PI3K or total Akt. Additionally, the activation of Akt phosphorylation was suppressed by using the Akt inhibitor, suggesting that MLE promoted the phosphorylation of Akt. These findings indicate that MLE or 1-DNJ supplementation could ameliorate insulin resistance by activating the IRS-1/PI3K/Akt signaling pathway.

### 3.8. Effects of MLE or 1-DNJ on Glycogen Synthesis in Diabetic Skeletal Muscle

**Figure 7:**
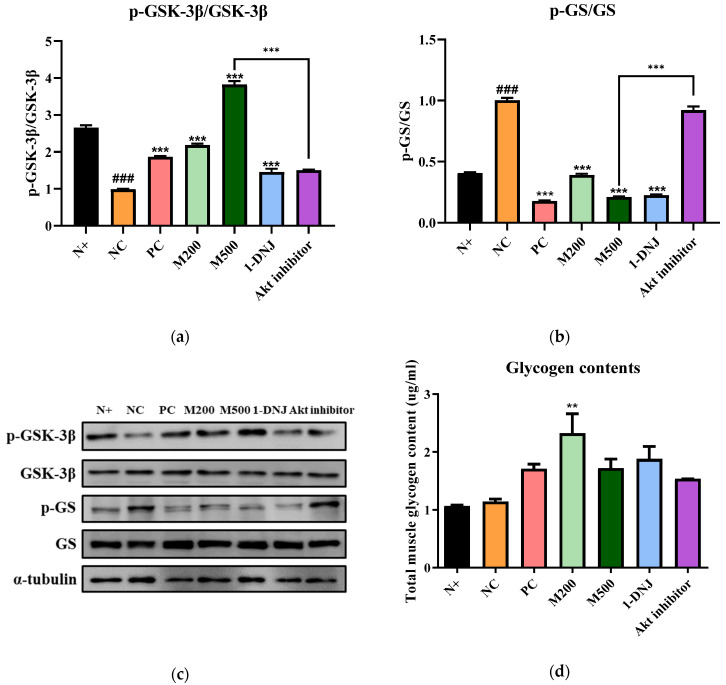
Effects of MLE or 1-DNJ on glycogen synthesis of diabetic skeletal muscle. (**a**) p-GSK-3β/GSK-3β. (**b**) p-GS/GS. (**c**) Western blot analysis for p-GSK-3β, GSK-3β, p-GS, and GS protein expression in diabetic skeletal muscle. (**d**) The contents of glycogen in skeletal muscle. N+, *db*/*m* + saline; NC, *db*/*db* + saline; PC, *db*/*db* + metformin 200 mg/kg/day; M200, *db*/*db* + MLE 200 mg/kg/day; M500, *db*/*db* + MLE 500 mg/kg/day; 1-DNJ, *db*/*db* + 1-DNJ 40 mM/kg/day; Akt inhibitor, *db/db* + MLE 500 mg/kg/day + Akt inhibitor 30 mM/kg/day. Values are expressed as mean ± SE. ### *p* < 0.001 vs. N+; ** *p* < 0.01, *** *p* < 0.001 vs. NC.

The expression level of p-GSK-3β was significantly lowered in the NC and Akt inhibitor groups, whereas MLE or 1-DNJ supplementation elevated the level of p-GSK-3β (Figure 7a). Compared with the N+ group, the expression levels of p-GS dramatically increased in the NC and Akt inhibitor group, but MLE or 1-DNJ supplementation reduced the GS phosphorylation (Figure 7b). Accordingly, the muscle glycogen content in the MLE- or 1-DNJ- treated groups increased, as compared with the NC group (Figure 7d). These findings indicate that MLE or 1-DNJ supplementation may regulate the expressions of GSK-3β and GS proteins, leading to the up-regulation of glycogen content in diabetic skeletal muscle.

## 4. Discussion

Many studies have found that mulberry leaf extract (MLE) is known to have therapeutic potential for numerous diseases, including diabetes, cardiovascular diseases, and cancer [21,23]. Furthermore, several studies have reported that MLE alleviates insulin resistance, and the active components of *Morus alba* L. have anti-hyperglycemic and anti-hyperlipidemic effects, suggesting a therapeutic effect on T2DM [24,25,26,27,28]. However, there have been no reports of the regulatory processes involving the IRS1/PI3K/Akt pathway in the skeletal muscle of leptin receptor-deficient *db*/*db* mice commonly used to study the T2DM mechanism. The previous study suggests that some of the disadvantages of polyphenols extracted directly from leaves could be cytotoxicity due to a low extraction yield, the retention of the solvents used for extraction, or the mobile phases used for the separation [29]. 

Depending on the various extraction methods, the function of the active ingredients may be different, and it may be a composite of many components [30,31]. Accordingly, we explored the active compounds in the lyophilized MLE powder by GC-TOF-MS and UHPLC-LTQ-Orbitrap-MS analyses. As shown in Tables S1 and S2, MLE consists of amino acids, sugar, sugar derivatives, fatty acids, and other functional components, including flavonols and lysophospholipids. Among the various ingredients, 1-DNJ is recognized for its ability to combat diabetes by primarily working as an alpha-glucosidase inhibitor [20,21]. Given that MLE and 1-DNJ both have anti-diabetic effects in this study, our study conditions may impact on 1-DNJ working as a main functional component of MLE. For the proper dose setting of MLE and 1-DNJ in vivo, we measured the toxicity of MLE and 1-DNJ in vitro (Figure S2), and then converted the in vitro concentration to the in vivo dose by referring to several studies [32,33,34]. 

Recently, researchers have reported that the GLUT4 in skeletal muscles is significantly associated with insulin resistance [35,36,37,38]. Our current study has shown that supplementation with MLE or 1-DNJ promotes the translocation of GLUT4 from an intracellular location to the membrane, suggesting that MLE or 1-DNJ may positively influence glucose uptake. In addition, PPARγ plays a crucial role in regulating glucose and lipid metabolism, and is well known to improve insulin sensitivity [39,40]. Furthermore, PGC-1α stimulates skeletal muscle mitochondrial biogenesis, regulates the pathophysiology of muscle, and is involved in the conversion of muscle type II glycolytic fibers to type I oxidative fibers [41]. Contrary to the liver, the expression of PGC-1α in diabetic muscle is down-regulated [42]. As expected, we confirmed that the *db*/*db* mice decreased the expression of PGC-1α, whereas the MLE or 1-DNJ treatment elevated the expression of PGC-1α, contributing to the regulation of GLUT4 in skeletal muscle. Studies suggest that PGC-1α is involved in disorders such as diabetes, and can be a therapeutic target to prevent the development of T2DM [42,43]. In this study, glucose utilization may have been ameliorated by raising the expression of PPARγ and PGC-1α, and accelerating the translocation of GLUT4 from the intracellular storage vesicles to the cell membrane in the skeletal muscle. Furthermore, MLE or 1-DNJ supplementation reduced further weight gain, decreased FBGLs, and improved glucose tolerance and insulin tolerance, as demonstrated by the AUC levels derived from the OGTT and ITT. In addition, the serum TG, TC, insulin concentrations, and HOMA-IR of mice supplemented with MLE or 1-DNJ were lower than in the NC group in diabetic *db*/*db* mice, which are well-established leptin receptor-deficient animal models characterized by lipid abnormalities. The use of *db*/*db* mice with a leptin receptor gene mutation model and the fact that they only assessed the weight reduction effect for a short time are both limitations of this study. Therefore, more research is required to evaluate the anti-obesity effect of MLE and 1-DNJ utilizing an obese mouse model or a long-term experimental period [44,45].

Skeletal muscle insulin resistance correlates with the occurrence of T2DM, and triggers significant structural, metabolic, and functional changes, such as muscle atrophy, fiber-type transition, impaired glucose uptake, and glycogen synthesis [46,47]. Since insulin-induced glucose uptake occurs in the skeletal muscle, the decline of muscle mass could reduce its capacity to take up glucose from the blood [48], and reduced muscle fiber size is associated with decreased insulin sensitivity [49,50]. Generally, T2DM is associated with muscle atrophy caused by IR, and defective leptin signaling may be toxic to early muscle growth in leptin receptor-deficient *db*/*db* mice [51]. The mechanism causing muscle atrophy in diabetes is unclear, but *db*/*db* mice exhibit reduced muscle fiber size and the deformation of muscle [52]. Although it is generally difficult to predict the amounts of active components of MLE delivered to skeletal muscle [29], this study observed packed and well-arranged muscle fibers in *db*/*db* mice on MLE or 1-DNJ supplementation compared to the NC group, which had muscle fibers that were deformed and irregularly arranged. Furthermore, the weight of the muscle increased in the M500 group compared with the NC group, and the muscle fiber size significantly increased with MLE or 1-DNJ supplementation, suggesting that they could help ameliorate IR. 

Glucose uptake into cells is initiated through the activation of IRS-1, which is the most critical signaling protein in the muscle, facilitates the cellular response to insulin [8], and its activation promotes phosphorylation of PI3K and Akt [53]. Furthermore, activating the PI3K/Akt pathway can induce skeletal muscle hypertrophy [54], defined as an increase in skeletal muscle mass, and can subsequently stimulate translocation of GLUT4 vesicles to the plasma membrane and thereby enhance glucose transport [14,55]. Western blot analysis has shown that the MLE or 1-DNJ treatment increases the protein expression of IRS-1-, p-PI3K-, and p-Akt-related insulin signal transduction, cell proliferation, metabolism, and apoptosis. The Akt inhibitor was used to confirm the activation of the IRS-1/PI3K/Akt pathway in skeletal muscle by MLE administration, and intraperitoneally injected before each 1 h FBGLs, OGTT, and ITT examination. As a result, the Akt inhibitor significantly influenced various organ and tissue levels, and several mice died during the experiment. It was difficult to produce significant results in this study. Similar to the previous study [56], our study also found that the Akt inhibitor with the MLE 500 mg/kg/day treatment could inhibit the IRS-1/PI3K/Akt signaling activity in western blot analysis. 

Glucose transport is the critical step for insulin-stimulated glycogen synthesis, which is dependent on the activity of the GS and glucose uptake [57]. Additionally, activation of the GS is mediated by GSK-3β, which contributes to glycogen synthesis, and is considered to be of importance in glucose homeostasis [17]. MLE or 1-DNJ supplementation promote glycogen storage in the skeletal muscle via the up-regulation of the activities of p-GSK-3β while down-regulating the p-GS levels. These results indicate that MLE or 1-DNJ supplementation improve IR via the activation of the insulin signaling pathway and glycogen synthesis in the skeletal muscle of *db*/*db* mice. 

## 5. Conclusions

In summary, we used *db*/*db* mice to evaluate whether MLE or 1-DNJ supplementation could improve muscle insulin resistance by modulating the expression of proteins related to the insulin signaling pathway and glucose metabolism. Accordingly, these results have shown that MLE or 1-DNJ may be considered alternative therapies for treating diabetic complications related to impaired glucose uptake, muscle fiber deformation, and mass muscle decline. Our findings present that MLE or 1-DNJ supplementation could recover the deformation of skeletal muscle. However, further studies are necessary to elucidate the detailed mechanism involved in this effect. 

## Data Availability

Not applicable.

## Supplementary Materials

The following supporting information can be downloaded at: https://www.mdpi.com/article/10.3390/life12101630/s1, Figure S1: Representative GC-TOF-MS chromatographic profiles of metabolites from a lyophilized MLE powder; Figure S2: Effects of MLE and 1-DNJ on the viability in beta cells. * *p* < 0.05 and *** *p* < 0.001 vs. CON; Table S1: Discriminative metabolites and their relative contents in lyophilized mulberry leave extract (MLE) powder using GC-TOF-MS; Table S2: Discriminative metabolites and their relative contents in lyophilized mulberry leaves extract (MLE) powder using UHPLC-LTQ-Orbitrap-MS/MS.
