# Supplementary material for: Mulberry (Morus alba L.) Leaf Extract and 1-Deoxynojirimycin Improve Skeletal Muscle Insulin Resistance via the Activation of IRS-1/PI3K/Akt Pathway in db/db Mice
Source: Life (Basel). 2022 Oct 18;12(10):1630. doi: 10.3390/life12101630 (PMC9604886; doi:10.3390/life12101630)
Supplement: Supplementary file 1 [file life-12-01630-s001.zip › life-1942481-supplementary/supplementary figures.pdf]

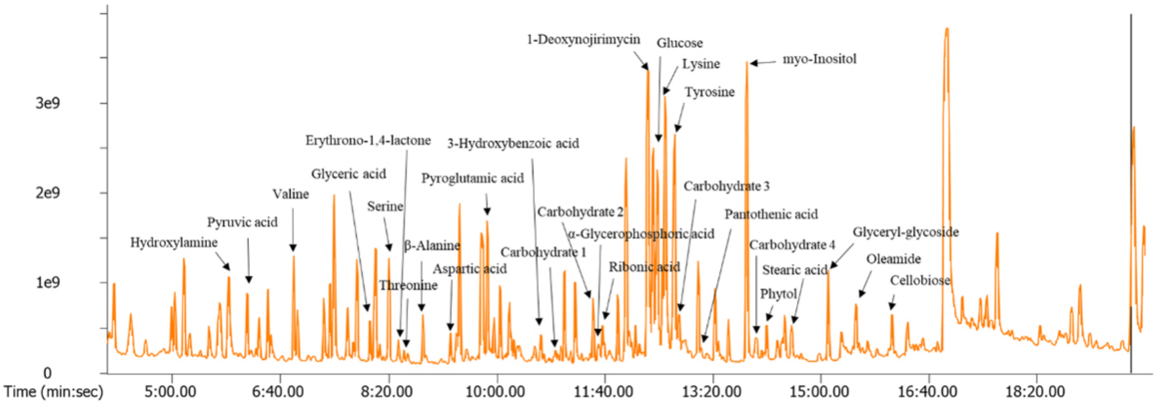

Figure S1. Representative GC-TOF-MS chromatographic profiles of metabolites from a lyophilized MLE powder.

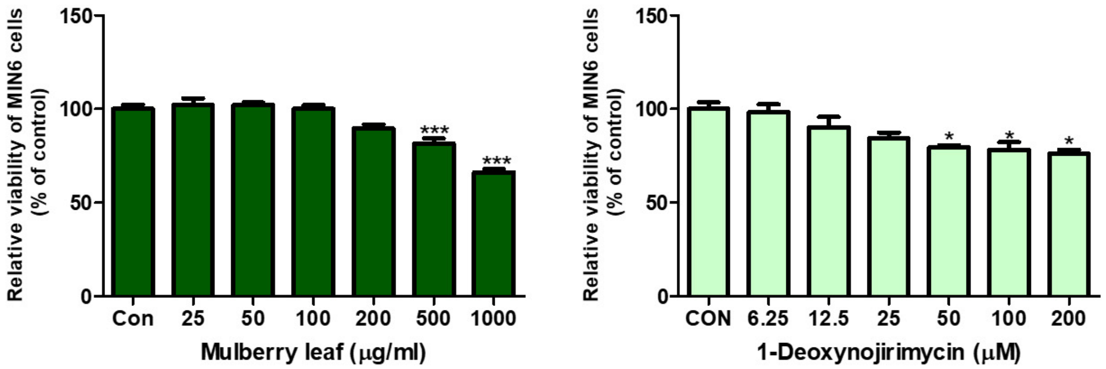

Figure S2. Effects of MLE and 1-DNJ on the viability in beta cells. * $p < 0.05$ and *** $p < 0.001$ vs. CON.
