# Supplementary material for: Mulberry (Morus alba L.) Leaf Extract and 1-Deoxynojirimycin Improve Skeletal Muscle Insulin Resistance via the Activation of IRS-1/PI3K/Akt Pathway in db/db Mice
Source: Life (Basel). 2022 Oct 18;12(10):1630. doi: 10.3390/life12101630 (PMC9604886; doi:10.3390/life12101630)
Supplement: Supplementary file 1 [file life-12-01630-s001.zip › life-1942481-supplementary/Table S1.pdf]

Table S1. Discriminative metabolites and their relative contents in lyophilized mulberry leave extract (MLE) powder using GC-TOF-MS.

No.	Tentative identifications[a]	tR[b] (min:sec)	Unique mass (m/z)	MS[n] fragment pattern (m/z)	TMS[c]	ID[d]
Amino acids						
1	Valine	06:55.8	144	144, 73, 218, 147, 145, 100, 59, 74, 75, 146	2	STD MS
2	Serine	08:19.3	204	73, 204, 218, 75, 205, 116, 74, 219, 206, 278	3	STD
3	Threonine	08:34.2	117	73, 218, 117, 219, 101, 147, 57, 75, 74, 291	3	STD
4	β-Alanine	08:54.6	174	174, 73, 248, 147, 86, 290, 100, 59, 133, 175, 249	3	MS
5	Aspartic acid	09:19.9	232	73, 232, 70, 147, 100, 75, 233, 133, 74, 59, 148	3	STD MS
6	Pyroglutamic acid	09:46.2	156	156, 73, 147, 157, 74, 230, 258, 72, 58, 75, 231	2	MS
7	Lysine	12:39.7	156	73, 174, 156, 317, 128, 318, 230, 100, 86, 175	4	STD MS
8	Tyrosine	12:48.1	218	218, 73, 219, 100, 280, 220, 179, 74, 281	3	STD MS
Sugar and Sugar derivatives						
9	Glyceric acid	08:02.8	189	73, 147, 189, 103, 133, 292, 102, 117, 74, 205	3	MS
10	Carbohydrate 1	10:59.7	103	73, 103, 147, 217, 205, 307, 117, 74, 133, 173	-	MS
11	Carbohydrate 2	11:29.4	205	73, 147, 205, 217, 117, 74, 148, 133, 394, 206	-	MS
12	Ribonic acid	11:43.7	103	73, 147, 103, 292, 217, 74, 205, 128, 189, 133	5	MS
13	Glucose	12:36.2	205	73, 205, 73, 319, 147, 160, 103, 217, 117, 320	5	STD MS
14	Carbohydrate 3	12:51.8	319	73, 147, 319, 205, 217, 74, 103, 117, 129, 204, 148	-	MS
15	myo-Inositol	13:51.5	217	73, 217, 147, 305, 191, 318, 306, 204, 218, 129, 319	6	STD MS
16	Carbohydrate 4	14:05.3	319	73, 319, 147, 205, 103, 74, 320, 133, 217, 117	-	MS
17	Glyceryl-glycoside	15:06.8	204	204, 73, 147, 217, 103, 205, 129, 206, 337	6	MS
18	Cellobiose	16:05.9	204	204, 73, 205, 147, 217, 117, 321, 103, 129, 206	8	MS
Fatty acids						
19	Stearic acid	14:32.9	117	117, 73, 75, 132, 129, 341, 145, 55, 342	1	STD MS
20	Oleamide	15:32.8	131	75, 131, 144, 73, 116, 128, 55, 338, 69, 353, 115, 145	1	MS
Etc.						
21	Hydroxylamine	05:52.1	133	73, 133, 146, 119, 59, 147, 249, 130	3	MS
22	Pyruvic acid	06:08.9	133	73, 147, 133, 59, 100, 72, 148, 220, 74, 86, 235	2	MS
23	Erythrono-1,4-lactone	08:29.3	101	73, 147, 75, 101, 59, 247, 103, 148, 102, 116	2	MS

24	Hydroxybenzoic acid	10:32.7	267	267, 223, 193, 282, 268, 126, 269, 224, 194, 283	2	STD MS
25	α-Glycerophosphoric acid	11:35.5	299	73, 299, 357, 147, 101, 103, 133, 211, 129	4	MS
26	1-Deoxynojirimycin	12:09.0	420	420, 216, 147, 421, 217, 422, 129, 133, 218	-	STD
27	Pantothenic acid	13:09.6	291	75, 291, 157, 117, 201, 247, 159, 129, 55, 144, 420	3	MS
28	Phytol	14:09.9	143	143, 73, 75, 144, 123, 57, 55, 81, 69, 103	1	MS
Non-Identifications						
29	N.I. 1	04:06.4	171	171, 73, 172, 78, 173, 64, 151, 186, 100	-	-
30	N.I. 2	04:34.0	89	73, 89, 59, 161, 74, 90, 60, 58, 75, 91	-	-
31	N.I. 3	05:02.0	123	123, 93, 55, 125, 95, 103, 59, 124	-	-
32	N.I. 4	06:17.2	86	86, 75, 73, 87, 74, 188, 146, 103, 70, 61, 170, 76	-	-
33	N.I. 5	06:31.0	86	86, 75, 73, 69, 87, 74, 146, 57, 56, 188, 103	-	-
34	N.I. 6	08:15.4	57	57, 73, 97, 127, 215, 111, 54, 109, 69, 216, 246, 159	-	-
35	N.I. 7	09:33.0	84	84, 75, 158, 157, 186, 73, 85, 56, 116, 103	-	-
36	N.I. 8	09:55.7	205	205, 292, 147, 220, 217, 102, 232, 293, 221, 142, 206, 149	-	-
37	N.I. 9	10:03.4	117	73, 147, 292, 117, 205, 217, 103, 74, 220, 133	-	-
38	N.I. 10	10:07.3	129	73, 129, 147, 247, 75, 203, 157, 85, 149, 133, 349	-	-
39	N.I. 11	10:41.1	55	73, 217, 55, 147, 75, 218, 129, 74, 221	-	-
40	N.I. 12	10:43.9	245	73, 245, 147, 231, 69, 74, 133, 246, 117	-	-
41	N.I. 13	10:56.9	129	73, 147, 75, 129, 74, 149, 133, 109, 247, 148, 363	-	-
42	N.I. 14	11:02.9	260	73, 144, 260, 147, 170, 129, 116, 75, 128, 145, 261		
43	N.I. 15	12:02.6	154	73, 288, 154, 147, 198, 244, 289, 82, 74		
44	N.I. 16	17:43.1	129	73, 129, 147, 217, 103, 361, 74, 169, 204, 191	-	-

[a] Tentative metabolites based on variable important projection (VIP) analysis with a cutoff value of 0.7 and $p < 0.05$. [b] Retention time; [c] Trimethylsilyl; [d] Identification.
