# Supplementary material for: Mulberry (Morus alba L.) Leaf Extract and 1-Deoxynojirimycin Improve Skeletal Muscle Insulin Resistance via the Activation of IRS-1/PI3K/Akt Pathway in db/db Mice
Source: Life (Basel). 2022 Oct 18;12(10):1630. doi: 10.3390/life12101630 (PMC9604886; doi:10.3390/life12101630)
Supplement: Supplementary file 1 [file life-12-01630-s001.zip › life-1942481-supplementary/Table S2.pdf]

Table S2. Discriminative metabolites and their relative contents in lyophilized mulberry leaves extract (MLE) powder using UHPLC-LTQ-Orbitrap-MS/MS.

No.	tR[a] (min)	Tentative identifications[b]	[M-H]⁻	M.W.[c]	MS[d] fragment pattern (m/z)	Molecular Formula	△ppm	REF[e]
Carboxylic acids								
1	0.92	Malic acid	133.0150	134	115,87>71	C4H6O5	5.289	[2]
2	1.05	Citric acid	191.0202	192	111>66	C6H8O7	2.639	[5], LIB
Hydroxybenzoic acids								
3	1.36	Pantothenic acid	218.1041	219	146>88>59	C9H17NO5	3.412	[4], LIB
4	1.44	Gentisoyl hexoside	315.0726	316	153>109	C13H16O9	1.507	[3,5]
Phenolic acids								
5	0.84	Quinic acid	191.0568	192	173,127,111,85,170>113,143	C7H12O6	3.343	[2]
6	3.65	*p*-Coumaric acid	163.0408	164	119	C9H8O3	4.248	[1,5]
7	3.85	Caffeic acid	173.0461	180	135>120,80	C9H8O4	-	[1,2]
8	3.87	Caffeoylquinic acid	353.0883	354	191,173>126,85	C16H18O9	1.288	[1], LIB
9	4.44	Coumaroylquinic acid	337.0936	338	191,163>172,126,85	C16H18O8	2.193	[5], LIB
Flavonols								
10	4.01	Quercetin-hexosyl hexoside	625.1419	626	463,301>300,445>178,150,273,270	C27H30O17	1.420	[1,5], LIB
11	4.03	Quercetin-rhamnosyl dihexoside	771.1993	772	609,300>301>270,178,150,255	C33H40O21	0.530	[6]
12	4.23	Kaempferol-rutinoside-hexoside	755.2051	756	593>285>257	C33H40O20	1.488	[2,5]
13	4.39	Kaempferol-malonyl dihexoside	695.1475	696	651,489>489,285>284,327	C30H32O19	1.465	[2]
14	4.58	Quercetin-rhamnose-hexose-rhamnose	755.2044	756	300>271,255>243,227	C33H40O20	0.442	[2]
15	4.72	Kaempferol O-rhamnosyl rutinoside	739.2111	740	575,284,255>339,393,429,309,547>311	C33H40O19	2.730	[9]
16	4.84	Rutin	609.1480	610	301>271,255>243,227	C27H30O16	3.123	[1,2], LIB
17	4.96	Isoquercitrin	463.0892	464	301>178,150,271,255>150	C21H20O12	2.161	[1]
18	5.03	Kaempferol-rhamnosyl hexoside	593.1517	594	285,284>255,277,211	C27H30O15	0.905	[1,5]
19	5.10	Quercetin-malonyl hexoside	549.0892	550	505>301,463>271,178,150,255	C24H22O15	1.160	[2]
20	5.19	Kaempferol-hexoside	447.0945	448	285>255>227	C21H20O11	2.674	[1], LIB
Lysophospholipids								
21	8.22	LysoPC (18:3)	562.3160	517	502, 277>233,259	C26H48O7NP	1.669	[15]
22	8.46	LysoPE(18:2)	476.2795	477	279>261>243, 233	C23H44NO7P	2.619	[15]
23	8.65	LysoPC (18:2)	564.3313	519	504>279>261	C26H50NO7P	1.078	[13,15]
24	8.76	LysoPE (16:0)	452.2792	453	255>237	C21H44NO7P	2.007	[15]
25	9.00	LysoPC (16:0)	540.3313	495	480>255>237	C24H50NO7P	1.126	[15]
26	10.07	LysoPC (18:0)	568.3629	523	508>283>265	C26H54NO7P	2.372	[16]
Etc.								
27	0.82	Maltose	341.1105	342	179,161>160,142,88	C12H22O11	4.501	[4]
28	6.63	9,12,13-TriHOME	329.23425	330	229, 311,171>211,292>183, 274	C18H34O5	2.742	[14]

[a] Retention time. [b] Tentative metabolites based on variable important projection (VIP) analysis with a cutoff value of 0.7 and $p < 0.05$. [c] Molecular weight. [d] MS^n fragment patterns detected in the negative ion mode. [e] Reference LIB, in house Library.
